# Response to Huang *et al.* Comments on Lu *et al.* Association between Self-Reported Global Sleep Status and Prevalence of Hypertension in Chinese Adults: Data from Kailuan Community. *Int. J. Environ. Res. Public Health* 2015, *12*, 488–503

**DOI:** 10.3390/ijerph120302903

**Published:** 2015-03-05

**Authors:** Kai Lu, Rongjing Ding, Qin Tang, Jia Chen, Li Wang, Changying Wang, Shouling Wu, Dayi Hu

**Affiliations:** 1 Department of Cardiology, The First Affiliated Hospital of Chongqing Medical University, Chongqing 400016, China; E-Mails: lukai2013@foxmail.com (K.L.); drchenjia@foxmail.com (J.C.); wangli514@sina.cn (L.W.); wangchangying04@163.com (C.W.); dayihu2014@163.com (D.H.); 2 Heart Center, Peking University People’s Hospital, No.11 South Xizhimen Avenue, Beijing 100044, China; 3 Department of Education and Science, China Medical Association, Beijing 100044, China; E-Mail: drtangqin@163.com; 4 Department of Cardiology, The Kailuan General Hospital, Hebei United University, No.57, East Xinhua Avenue, Tangshan 063001, China

We thank Huang *et al.* [1] for their interest in reading our article and their time in writing their comments on our work [2]. Our response to their concerns are as follows:

Although the sample in our study has a significant sex ratio heterogeneity, we do not think it has an obvious bias on the results. You need to note that the association between sleep status and hypertension in Chinese males and females was assessed separately. Even though the sample size of the female is not as large as that of the male, it was sufficient to reach a reasonable conclusion.

Further, although the socioeconomic status is associated with the risk of hypertension just as the authors mentioned, we do not think this is the key point that leads to the differences between ours and other previous studies. We did not provide basic characteristics of the enrolled subjects with regard to their socioeconomic status, as most of the subjects are coal miners and therefore have a similar income. Actually, we think the main reasons accounting for discrepancies between ours and other previous studies may be that it is not sufficient to assess sleep only by sleep quality or sleep duration [3,4]. Research that we are currently conducting suggests that sleep duration and sleep quality have an additive effect on the risk of hypertension. Considering the fact that sleepers with short sleep duration often have a high prevalence of poor sleep quality, it is necessary to preclude the potential confounding effect of each, and confirm the specific and separate role of sleep quality and sleep duration on hypertension.
